# A Molecular Docking Approach to Evaluate the Pharmacological Properties of Natural and Synthetic Treatment Candidates for Use against Hypertension

**DOI:** 10.3390/ijerph16060923

**Published:** 2019-03-14

**Authors:** Syed Awais Attique, Muhammad Hassan, Muhammad Usman, Rana Muhammad Atif, Shahid Mahboob, Khalid A. Al-Ghanim, Muhammad Bilal, Muhammad Zohaib Nawaz

**Affiliations:** 1 Department of Computer Science, University of Agriculture, Faisalabad 38040, Pakistan; awais000936@gmail.com (S.A.A.); m.hassan2381996@gmail.com (M.H.); musmansahnigk@gmail.com (M.U.); 2 Department of Plant Breeding and Genetics, University of Agriculture, Faisalabad 38040, Pakistan; dratif@uaf.edu.pk; 3 Center for Advanced Studies in Agriculture and Food Security, University of Agriculture, Faisalabad 38040, Pakistan; 4 Department of Zoology, College of Science, King Saud University, P.O. Box 2455, Riyadh 11451, Saudi Arabia; shahidmahboob60@hotmail.com (S.M.); kghanim@ksu.edu.sa (K.A.A.-G.); 5 School of Life Science and Food Engineering, Huaiyin Institute of Technology, Huaian 223003, China; bilaluaf@hotmail.com

**Keywords:** angiotensin-converting enzyme, ligands, hypertension, molecular docking, drug designing

## Abstract

Cardiovascular diseases (CVDs) have become the leading cause of disability and death worldwide, particularly in low- and middle-income countries. Hypertension, a major cause of CVD progression, is widely attributable to genetic, behavioral, and environmental risk factors. Among the genetic reasons, angiotensin II enzyme, produced as a result of abnormal functioning of the renin–angiotensin system, is reported as the foremost cause of hypertension. A cascade of genes, including those encoding for WNK kinases (WNK1 and WNK4), Bp1, Bp2, angiotensinogen, and other enzymes, is involved in the conversion of angiotensin I to angiotensin II. However, the angiotensin-converting enzyme (ACE) plays a crucial role in this pathway. Therefore, ACE could be a potential therapeutic target in regulating the conversion of angiotensin I to angiotensin II and eventually controlling hypertension. In this study, a molecular docking-based approach was utilized for identifying and evaluating potential inhibitors of ACE present in herbs, other natural sources, and synthetic sources, on the basis of these compounds’ binding affinities and other physicochemical features. In addition, the suitability of these inhibitors as drugs for biological systems, considering their adsorption, distribution, metabolism, and excretion (ADME), was predicted using Lipinski’s rule. In conclusion, our study provides a novel and clearer insight into the interaction properties of known putative inhibitors of ACE.

## 1. Introduction

Cardiovascular diseases (CVDs) refer to disorders of the heart and blood vessels, including coronary artery diseases (CAD) such as angina and myocardial infarction, that are very common and represent a major challenge to sustainable human development nowadays [1]. With the turn of the century, these diseases have become the leading cause of disability and death around the globe, particularly in developing countries. Global status reports have shown that approximately 12.3 million deaths (25.8% of all deaths) globally were caused by CVDs in 1990 and increased to 17.9 million deaths (32.1% of all deaths) in 2015 [2]. In the United States, 11%, 37%, 71%, and 85% of people are affected by CVDs between 20 and 40, 40 and 60, 60 and 80, and over 80 years of age, respectively [3]. Risk factors of CVDs include genetic, behavioral, and environmental factors, such as high blood pressure, smoking, diabetes, physical inactivity, obesity/overweight, high blood cholesterol, low socioeconomic status, and excessive alcohol. Among these, high blood pressure is the main risk factor for CVD deaths (13%), followed by the tobacco (9%), diabetes (6%), lack of exercise (6%), and obesity (5%), and the prevalence of risk factors fluctuates in various regions [4].

Hypertensive heart disease mostly known as hypertension, refers to a group of disorders including heart failure, ischemic heart disease, and left ventricular hypertrophy and is becoming a major cause of death associated with high blood pressure worldwide [5]. Several genes including WNK1, WNK4, Bp1, Bp2, AGT, and ACE were reported to be involved in hypertension [6]. Mutation in WNK1 and WNK4 genes could cause disturbances in the homeostasis of K^+^, salts, and pH level [7], whereas a mutation in AGT genes present on chromosome 1 results in an imbalance of angiotensinogen production, ultimately leading to hypertension [8]. It was revealed that ACE plays a key role in hypertension, its dysfunction being the most frequent cause of hypertension [9]. The most comon biological reason behind hypertension is the production of angiotensin II enzyme, which is produced from the conversion of angiotensin I by the action of a series of the enzymes [10]. Therefore, regulating the conversion of angiotensin I to angiotensin II could be an effective strategy to control hypertension.

Angiotensin-converting enzyme (ACE) is considered crucial in this pathway and has received considerable attention as a therapeutic target for controlling hypertension. Repressing ACE expression has been proved as an effective strategy in controlling hypertension, as its downregulation will inhibit the conversion of angiotensin I to angiotensin II [11]. In this study, the binding affinities of various natural, synthetic, and herbal inhibitors for active sites of ACE were predicted using the molecular docking approach, which is becoming an extremely important tool in drug design. Molecular docking is playing a major role in structure-based molecular biology and computer-based drug design. The molecular docking methodology can be utilized to demonstrate the cooperation between a small molecule and a protein at the nanoscale, which empowers us to describe the behavior of small particles in the binding site of the proteins and explain key biochemical processes. Furthermore, drug-likeness and compatibility with gastrointestinal and brain absorption were computed for all the inhibitors tested to evaluate their suitability as potential therapeutic agents and orally active drugs for the treatment of hypertension.

## 2. Materials and Methods

### 2.1. Physiochemical Properties

The physiochemical properties of human ACE were predicted using Protparam [12]. The Protparam tool works on the basis of the Edelhoch method [13], determining the weight value of instability with respect to 400 different dipeptides (DIWV) and the hydropathy values for extinction coefficients, instability index (II), and GRAVY value (grand average of hydropathy value).

### 2.2. Secondary Structure Predictions

The number of helix turns and coils was calculated using “Psipred” [14]. Psipred used two feed-forward neural networks which perform an analysis of output obtained from PSI–BLAST (Position-Specific Iterated–BLAST) for secondary structure prediction.

### 2.3. Domain and Motif Analysis

Domains of human ACE were retrieved from the NCBI conserved domain database (https://www.ncbi.nlm.nih.gov/Structure/cdd/wrpsb.cgi?), and motifs were predicted using the MEME [15] software. To overcome the limitation of finding gapped motifs, an efficient algorithm of MEME motif discovery is integrated with the algorithm of GLAM2 [16] which enables users to discover novel motifs having gaps.

### 2.4. Selection of Ligand, Receptor, and Active Site Prediction

**Figure 1:**
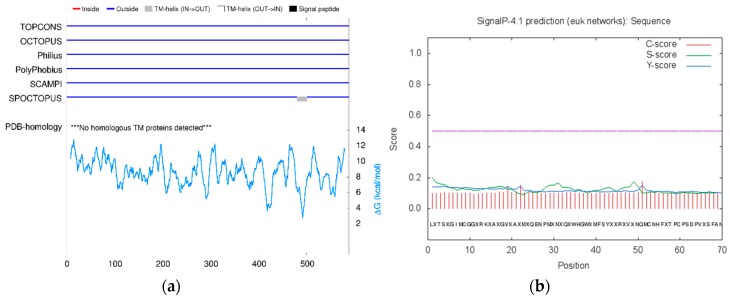
Results from (**a**) TOPCONS and (**b**) Signalp4 showed that ACE is present outside of the cell membrane and has no signal peptide.

The structures of ligand molecules were downloaded from PubChem that selects compounds based on their chemical formula and physiochemical properties [17]. To explore the binding sites of ligands (inhibitors) on the ACE structure, its 3D-structure was retrieved from RCSB-PDB (Figure 1). Active sites of human ACE were retrieved from InterPro (https://www.ebi.ac.uk/interpro/).

### 2.5. Preparation of Ligands and Receptor

Ligands and receptor were prepared for docking by minimizing their energy and then 3D protonating in MOE 2009.10 [18] by removing solvent molecules (water) and other sites on ACE, facilitating the interaction of only inhibitors or ligands with the selected receptor.

### 2.6. Molecular Docking

The scope of therapeutic targets in drug design and discovery has broadened with the completion of the human genome project. At the same time, advanced strategies such as excessive-throughput crystallography, protein purification, and nuclear magnetic resonance (NMR) spectroscopy have been providing structural information of protein–ligand and protein complexes [19,20]. All these advancements have resulted in the development of computer-aided drug design, also known as molecular docking. Molecular docking consists of structure-based and ligand-based methods. The ligand-based methods such as QSAR (quantitative structure–activity relationship) are preferred when information about a ligand is large; otherwise, structure-based docking methods are used [21]. In this study, molecular docking of reported synthetic and natural inhibitors of ACE was performed using diverse computational tools, with the aim to discover the optimum inhibitor, which ultimately would provide the basis for designing drugs against hypertension by inhibiting ACE. The structure-based docking method was used because structure-based Computer Aided Drug Designing (CADD) relies on the knowledge of the target protein structure to calculate interaction energies for all compounds tested, whereas ligand-based CADD exploits the knowledge of known active and inactive molecules through chemical similarity searches or construction of predictive, QSAR models [22]. Structure-based CADD is generally preferred where high-resolution structural data of the target protein are available, i.e., for soluble proteins that can readily be crystallized. These advancements in research allow computational techniques to analyze all factors involved in drug design and discovery.

A plethora of computational tools have been developed and are widely used for protein-ligand docking, particularly for discovering drugs based on small well-structured molecules. These tools include Glide, Gold, Auto dock, PyRx, Surflex, ICM, FITTED, MOE (Molecular Operating Environment). MOE, which helps to visualize, characterize, and evaluate protein interactions with other proteins or ligands, was utilized in this study. Proteins and small molecules can be designed using modern *in silico* design applications, and structure–activity relationships (SAR) in micro-molecules can be developed. It works on the basis of high-throughput screening, docking, energy determination, combining biology, chemistry, and information technology [23]. A list of widely used docking tools and their description are presented in Table 1.

MOE was selected for docking among various available resources as it has a user-friendly graphical interface. It represents a good graphical view of results by showing ligand and receptor binding residues with their positions and interactions. In MOE, receptor–ligand binding affinities with all possible binding geometries are prioritized on the basis of a numerical value called S-score. MOE has multi-disciplinary applications, such as in structure-based design, fragment-based design, pharmacophore discovery, medicinal chemistry applications, biologics applications, protein and antibody modeling, molecular modeling and simulations, cheminformatics and QSAR, and methods’ development and deployment. MOE identifies salt bridges, hydrogen bonds, hydrophobic interactions, sulfur-LP, cation-π, and solvent exposure, and gives the S score. Interactions of inhibitors with receptor proteins are predicted on the basis of the S score [24].

### 2.7. Lipinski’s Rule of Five for Drug-Likeness or ADME (Absorption, Distribution, Metabolism, and Excretion) Analysis

Drug-likeness of our inhibitors, including absorption, distribution, metabolism, and excretion of these inhibitors within the body, was predicted using SwissADME (Swiss Institute of Bioinformatics, Switzerland) [39]. The Egan BOILED-Egg method available in SwissADME tool was used for the determination of the absorption of the inhibitors in the gastrointestinal tract and brain. BOILED-Egg (Brain Or IntestinaL EstimateD permeation predictive model), also called Egan egg, provides a threshold (WLOGP ≤ 5.88 and TPSA ≤ 131.6) and a clear graphical representation of how far a molecular structure is from the ideal one for good absorption [40]. In this 2D graphical representation, the yolk area represents the molecules that can passively permeate through the blood–brain barrier (BBB), whereas the molecules located in the white region are predicted to be passively absorbed by the gastrointestinal (GI) tract.

Herein, we particularly focused on ACE, which has been reported as a key enzyme in hypertension [9]. Various substances containing naturally occurring compounds from herbal and animal sources (e.g., Allicin and teprotide) have come forward as potential inhibitors of ACE (Table 2) and may control high blood pressure.

## 3. Results and Discussion

### 3.1. Physiochemical Properties of ACE

Human ACE was found to exhibit a molecular weight of 67,993.2 Daltons and isoelectric pH 5.82. It is a stable protein with an aliphatic index of 78.86, whereas its instability index was predicted to be 39.46. The prediction of GRAVY value of −0.4441 demonstrates that ACE is a hydrophilic peptide (Table 3).

### 3.2. Membrane Topology of ACE

**Figure 2:**
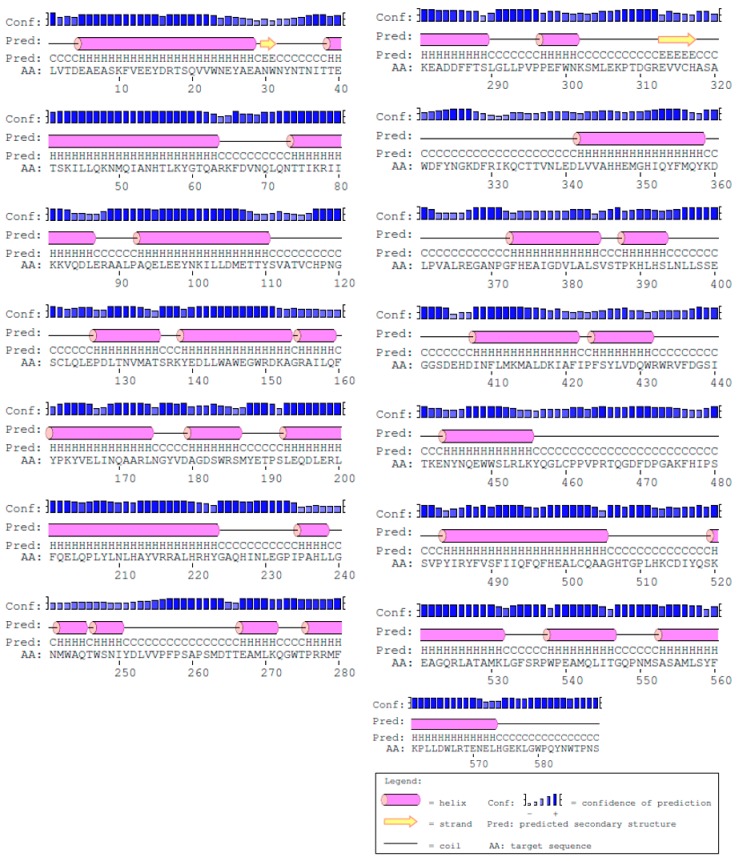
Secondary structure of human ACE; pink cylinders, yellow arrows, and black lines show helixes, strands, and coils, respectively.

**Figure 3:**
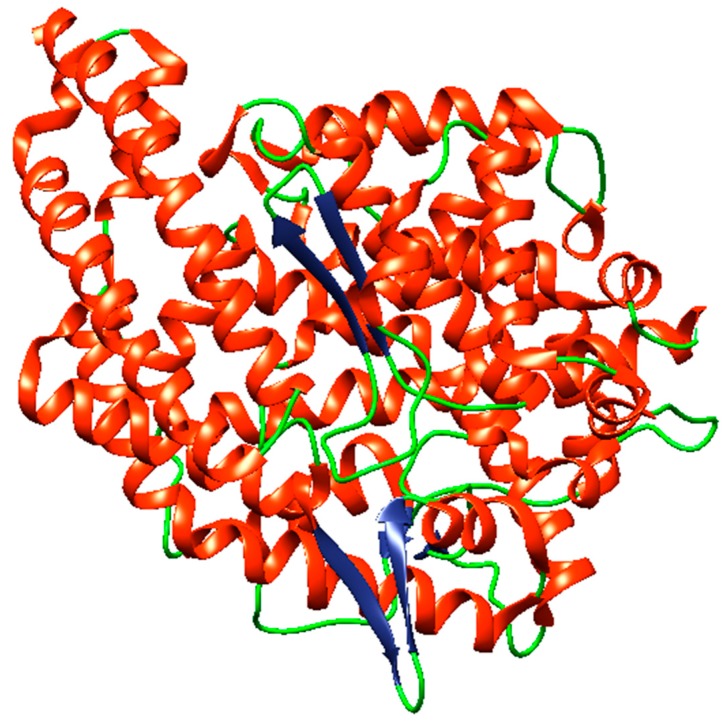
3D structure of human ACE (PDB ID: 1o8A), visualized through CHIMERA.

The use of diverse computational tools including TOPCON, Signal p4.1, OCTOPUS, PolyPhobius, SCAMPI, and SPOCTOPUS predicted that ACE is an extracellular enzyme, present outside of the cell membrane (Figure 1). The structure of human ACE was predicted to comprise 333 helices, 9 strands, and 247 coils (Figure 2). NCBI Conserved Domain Database has shown that human ACE has only one domain and belongs to the family Peptidase M2, peptidyl-dipeptidase A, with the interval from 11 to 571 amino acid numbers. However, MEME predicted that ACE has the sequences “VCHPNGSC” in position 115–122, “HHEMGHIQYFMQYK” at 346–359, and “FHEALC” at 373–378. The 3D structure of human ACE was retrieved from the PDB (PDB ID: 1o8A) online protein database and visualized with a desktop tool, i.e., CHIMERA (Figure 3). ACE was predicted to have 14 active sites for interactions using InterPro (EMBL-EBI, Cambridgeshire, UK) (Table 4).

### 3.3. Reported Inhibitors of ACE

**Figure 4:**
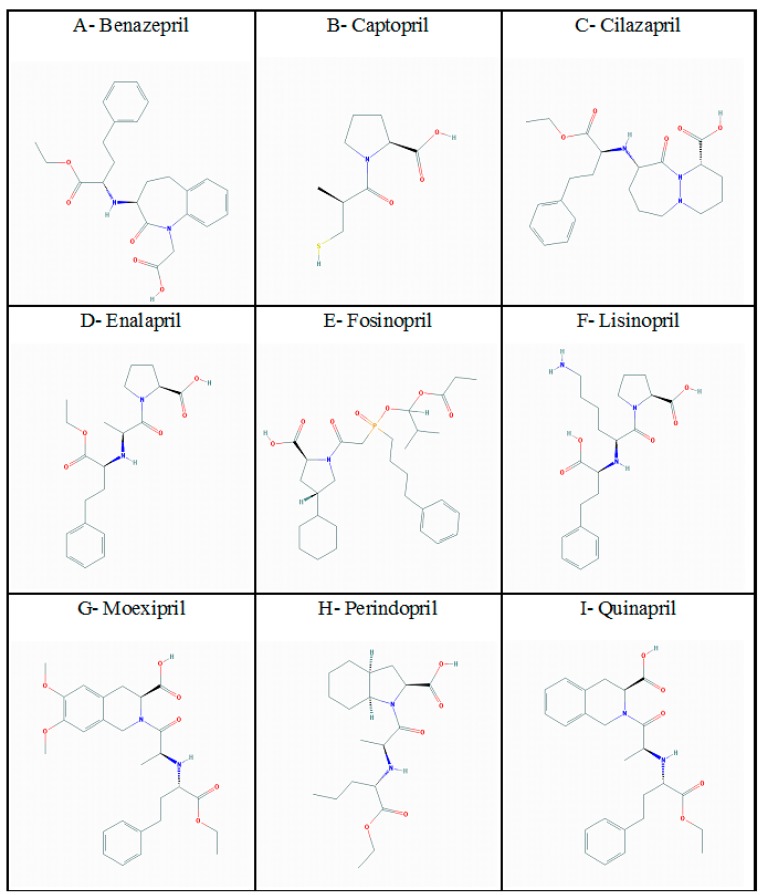

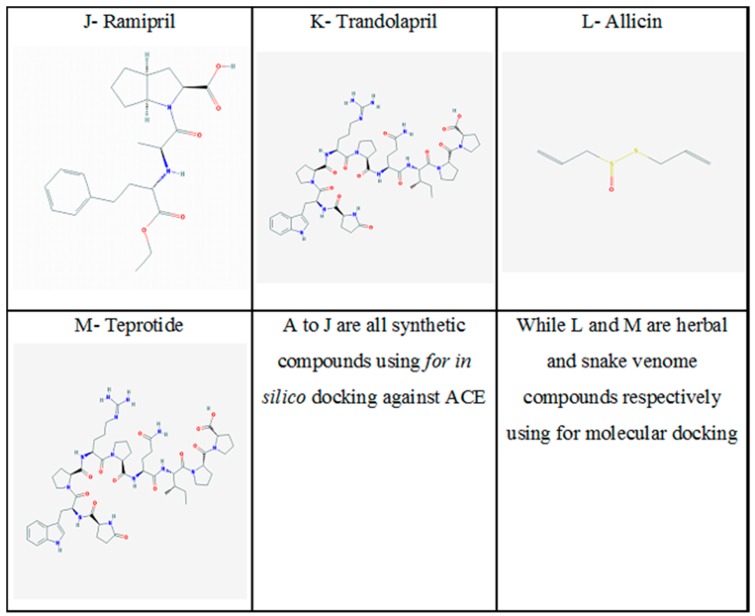
2D structures of various ACE inhibitors including (**A**) Benazepril, (**B**) Captopril, (**C**) Cilazapril, (**D**) Enalapril, (**E**) Fosinopril, (**F**) Lisinopril, (**G**) Moexipril, (**H**) Perindopril, (**I**) Quinapril, (**J**) Ramipril, (**K**) Trandolapril, (**L**) Allicin, and (**M**) Teprotide.

The 2D structures of reported inhibitors of ACE were downloaded from PubChem in SDF format and are portrayed in Figure 4.

Herbal and Natural Inhibitors

**(1)**  Garlic contains “allicin”, a chemical compound with a reported role in ACE inhibition.
  A fresh clove of garlic (4 g) contains about 1% allicin [54].
**(2)**  Snake (*Bothrops jararaca*) venom contains “teprotide”, which is known to have ACE inhibition activity [53].

### 3.4. Molecular Docking

In silico docking of human ACE against selected inhibitors was performed using MOE against all the predicted active sites. The results showed that all selected inhibitors were in the pocket of the target protein (ACE), exhibiting a possible interaction with ACE. The docking results were manipulated using the GBVI/WSA dG scoring function with the generalized Born solvation model (GBVI). The GBVI/WSA dG is a force field-based scoring function, which estimates the free energy of binding of the ligand from a given orientation. Interaction results were evaluated with the S score. Inhibitors with the lowest S score tend to establish a strong interaction with ACE on specific active sites (Table 5). After in silico docking, we identified a ligand showing the minimum S score among all the inhibitors. Teprotide, which is present in snake venom, showed a minimum S score of −20.1163; therefore, it establishes the strongest interaction with ACE among all the inhibitors discussed in this study. Fosinopril is another widely used and effective drug against hypertension. It was predicted to exhibit a strong binding affinity for ACE, with an S score of −18.9225. Earlier studies demonstrated that fosinopril doses of 10 and 20 mg could inhibit 85% and 93% of ACE activity, respectively, within 24 h of administration [55]. Hayek et al. used ACE as a receptor and fosinopril as an inhibitor to cure hypertension and concluded, after 12 weeks of treatment, that fosinopril remarkably reduces blood pressure in mice [56]. Heart Outcomes Prevention Evaluation Study Investigators evaluated the role of ramipril in reducing the overactivity of ACE and showed that ramipril significantly lessens the rates of myocardial infarction and stroke in a wide range of high-risk patients [57].

### 3.5. Drug-Likeness and ADME Predictions of Our Inhibitors

The antagonistic interaction of inhibitors with a receptor protein or enzyme cannot guarantee the suitability of an inhibitor as a drug; therefore, ADME analysis of inhibitors is important in the drug development [58]. ADME is based on Lipinski’s rule of five [59] and helps to make decisions on the approval of inhibitors for biological systems. Poor ADME characteristics and unfavorable toxicology for a biological system are the major cause of the failure of most medicines in clinical experiments. 

**Figure 5:**
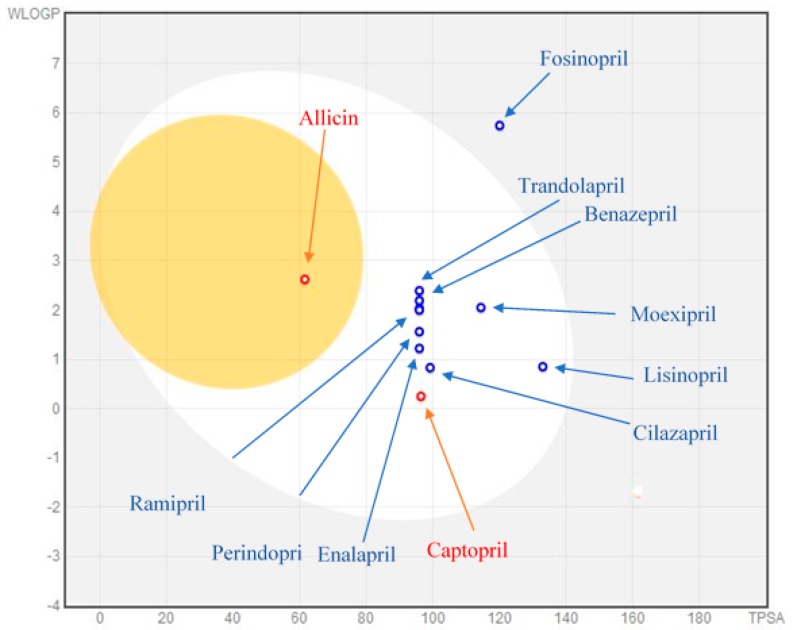
Evaluation of the analyzed ligands by the BOILED-Egg method.

The Lipinski’s rule of five was published in 1997 by Christopher A. Lipinski and is also known as the Pfizer’s rule of five or Rule of five (Ro5). It is a rule of thumb to evaluate the drug-likeness and to determine if a chemical compound with a certain pharmacological or biological activity has properties that would make it a likely orally active drug in humans. Ro5 depends on four simple physiochemical parameter ranges: the molecular weight (MW), which should be less than 500 g/mol, lipophilicity (Log *P*) less than 5, and number of hydrogen bond donors and acceptors less than 5 and 10, respectively, as seen for 90% of orally functional drugs that have obtained phase II clinical status. These parameters are connected with intestinal permeability and aqueous solubility and determine the first step of oral bioavailability. These rules explain molecular properties valuable for a drug’s pharmacokinetics in the human body, including their absorption, distribution, metabolism, and excretion (ADME). If a ligand fails to fulfill the parameters of Ro5, then it is highly probable that it will cause trouble if ingested [1]. ADME predictions of our inhibitors are shown in Table 6. BOILED-egg results, showing the possibility of absorption and penetration of inhibitors in the GI attract and brain using WLOGP and TPSA parameters are presented in Figure 5.

All of the inhibitors or ligands discussed herein satisfy the Lipinski’s rule, except for teprotide, which significantly violates three parameters (MW > 500, number of hydrogen bond donors > 5 and number of hydrogen bond acceptors > 10); furthermore, it also violates the BOILED-egg method. Although teprotide has the highest binding affinity for human ACE among all the inhibitors, it is not proposed as an orally active drug due to violation of the Lipinski’s rule. An Egan’s egg graph for the inhibitors was generated using SwissADME. The graph showed that only allicin, a herbal compound, is absorbed by the brain, though in the acceptable range. The remaining inhibitors showed gastrointestinal absorption within an acceptable range, except for teprotide and lisinopril (WLOGP >5.88 and TPSA >131.6) (Figure 5).

So, on the basis of Egan’s boiled-egg rule threshold values (WLOGP ≤ 5.88 and TPSA ≤ 131.6), only allicin penetrates the blood–brain barrier, though within acceptable limits. The blue dots indicate molecules predicted to be effluated from the CNS by P-glycoprotein, and the red dots indicate molecules predicted not to be effluated from the CNS by P-glycoprotein.

The above-mentioned results of the molecular docking that were obtained using molecular operating environment (MOE) allowed us to observe ligand–receptor. Analysis of the interactions with the Protein–Ligand Interaction Profiler between ACE and the inhibitor teprotide revealed the best binding affinity. By analyzing the drug’s score (S-value), teprotide showed the lowest S-value (−20.1163), resulting as the best ligand among our selected ligands to inhibit the activity of ACE. However, it violates three parameters (MW > 500D, H-bond donors >5 and H-bond acceptors >10) of the Lipinski’s rule, as well as Egan’s parameters (WLOGP > 5.88 and TPSA > 131.6). Although teprotide is proposed to be a potential therapeutic inhibitor of ACE, it may fail as an orally active drug because it deviates from the Lipinski’s rule and from the Egan’s rule. Compared to teprotide, the higher S-value (−18.9225) of fosinopril demonstrates its lower binding affinity for ACE. Notably, fosinopril satisfies all parameters of the Lipinski’s rule, except for MW >500D, and also complies to the BOILED-egg approach, showing no brain and GI tract absorption, which renders it a more suitable potential orally active drug and therapeutic inhibitor of ACE to be tested in clinical trials, compared to teprotide. Allicin is a herbal compound found in garlic that helps to inhibit the activity of ACE and satisfy all Lipinski’s rules with minimal absorption in the brain. Nonetheless, it displayed lower binding affinity and placed last among our selected inhibitors because of its highest S-value (−5.5448). Although allicin has a lowest binding affinity for ACE, it can be used in drug design for the treatment of hypertension because of its herbal nature; also, garlic can be used as food to bring the blood pressure within normal range. Therefore, from various investigations, it is clear that teprotide, which is extracted from a snake (*B. jararaca*) venom, has the highest binding affinity for ACE compared to other inhibitors but it cannot be used as an orally active drug. In contrast, fosinopril, a synthetic compound, showed the second highest binding affinity for ACE and therefore could be used as a potentially therapeutic compound for the development of orally active drugs inhibiting the activity of ACE and thereby useful to treat hypertension. Fosinopril also does not exhibit any type of absorption in the brain and gastrointestinal tract. Similarly, allicin can also be used to develop orally active drugs for the management of hypertension. 

## 4. Conclusions

In this study, fosinopril was predicted as the best ACE inhibitor (with maximum binding affinity for ACE after teprotide) to be used as a potentially therapeutic orally active drug (on the basis of Lipinski’s rule of five and BOILED-egg approach) for the treatment of hypertension. Among the animal inhibitors, teprotide showed the highest binding affinity compared to all other ligands studied here; however, according to Lipinski’s rule and BOILED-egg method, it is not recommended as a suitable therapeutic agent. Furthermore, allicin, a herbal ligand, exhibited reasonable binding affinity for ACE and follows Lipinski’s rule of five but can only be used as food because of its slight absorption in the brain. In conclusion, our study provides a clearer insight into the interaction properties of known putative synthetic inhibitors of ACE and bioactive inhibitors, including interactions with the blood–brain barrier. In recent years, consumers have paid attention to natural bioactive compounds as potential medicines because of their effectiveness in promoting health, associated with less adverse effects. In future, we will be able to use the knowledge of inhibitors’ pharmacological properties, including those of bioactive compounds such as allicin, to make effective therapeutic drugs based on ACE inhibition to cure hypertension.

## Figures and Tables

**Table 1:** A list of widely used tools for docking.

No.	Software/Tools	Algorithm	Scoring Term	Advantages	Ref.
1.	Molecular Operating Environment (MOE)	High-Speed α shapes algorithms	London dG, FlexX, DrugScore, Mcdock	Customizable, available source-code, gives binding affinity score, shows interacting amino acids with position, and is user-friendly.	[25]
2.	PyRx	Lamarckian genetic algorithm	Binding energy, Internal energy, Internal energy, Unbound energy	Temperature Resistance. Pyrex’s excellent thermal properties at both high and low temperatures are one of its key features.	[26]
3.	Glide (Grid-based Ligand Docking with Energetics)	Monte Carlo	Glide score	Lead discovery and lead optimization	[27]
4.	AutoDock	Lamarckian genetic algorithm	Empirical free-energy function	Adaptability to user-defined input	[28]
5.	GOLD (Genetic Optimization for Ligand Docking)	Genetic algorithm	GoldScore, ChemScore, ASP (Astex Statistical Potential), CHEMPLP (Piecewise Linear Potential), User-defined	Allows atomic overlapping between protein and ligand	[29]
6.	Surflex	Surflex-Dock search Algorithm	Bohm’s scoring function	High accuracy level by extending force fields	[30]
7.	FlexX	Incremental reconstruction	Modified Bohm scoring function	Provides a large number of conformations	[31]
8.	ICM (Internal Coordinate Modeling)	Monte Carlo minimization	Virtual library screening scoring function	Allows side chain flexibility to find a parallel arrangement of two rigid helixes	[32]
9.	MVD (Molegro Virtual Docker)	Evolutionary algorithm	MolDock score	High accuracy level of predicting binding mode	[33]
10.	Fred (Fast Rigid Exhaustive Docking)	Exhaustive search algorithm	Gaussian scoring function	Nonstochastic approach to examine all possible poses within a protein active site	[34]
11.	LigandFit	Monte Carlo method	LigScore, Piecewise Linear Potential (PLP), Potential of Mean Force (PMF)	Generates good hit rates based on LigScore	[35]
12.	FITTED (Flexibility Induced Through Targeted Evolutionary Description)	Genetic algorithm	Potential of Mean Force (PMF), Drug Score	Analyzes the effect of water molecules on protein–ligand complexes	[36]
13.	GlamDock	Monte Carlo method	ChillScore	Provides provision of two-dimensional analysis to screen ligands by targeting protein	[37]
14.	iGEMDOCK	Genetic algorithm	Empirical scoring function	Integrates the structure-based virtual screening and post-screening analysis. Provides a graphical integrated environment for virtual screening	[38]

**Table 2:** Synthetic, herbal, and animal source inhibitors of angiotensin-converting enzyme (ACE).

No.	Ligand	Features	Source	Function	Citation
1.	Benazepril	97% protein binding, a half-life of 10–11 h, pregnancy category: D	Synthetic	Cures hypertension	[41]
2.	Captopril	25–30% protein binding, a half-life of 2 h, pregnancy category: D	Synthetic	Controls blood pressure	[42]
3.	Cilazapril	A half-life of 1 to 4 h	Synthetic	ACE inhibition	[43]
4.	Lisinopril	Pregnancy category: D, does not bind serum proteins other than ACE	Synthetic	Inhibition of ACE	[44]
5.	Moexipril	Pregnancy category: D, <90% protein binding and 1 h half-life	Synthetic	Treatment of hypertension and congestive heart failure	[45]
6.	Trandolapril	Half-life 6 to 10 h, pregnancy category: D	Synthetic	Controls high blood pressure	[46]
7.	Enalapril	Pregnancy category: D, half-life of 11 h	Synthetic	ACE inhibition to control hypertension	[47]
8.	Fosinopril	12 h half-life, pregnancy category: D, ≥95% protein-binding capacity	Synthetic	Normalizes blood pressure	[48]
9.	Perindopril	20% protein binding, pregnancy category: D and 1–2 h half life	Synthetic	Controls blood pressure	[49]
10.	Quinapril	97% protein binding, 2 h biological half-life, pregnancy category: D	Synthetic	Inhibition of ACE	[50]
11.	Ramipril	Protein binding 73% (ramipril), 56% (ramiprilat), half-life of 2–4 h	Synthetic	Congestive heart failure control	[51]
12.	Allicin	Has water solubility of 24 mg/mL at 10 °C, solid, melting point >25 °C	Garlic and onion	Inhibition of ACE	[52]
13.	Teprotide	Has 10 hydrogen bond donors, 13 hydrogen bond acceptors, and 79 heavy atoms	Snake venom	Antihypertensive agent	[53]

**Table 3:** Physiochemical properties of ACE predicted by ProtParam.

Serial Number	Property	Value
1.	Number of amino acids	589
2.	Total number of atoms	9457
3.	Molecular weight	67,993.20 Dalton
4.	Theoretical pI	5.82
5.	Extinction coefficient *	143,240 at Abs 0.1% 2.112, assuming all pairs of Cys residues form cystines
6.	Instability index	39.46
7.	Aliphatic index	78.86
8.	Grand average of hydropathicity (GRAVY)	−0.441
9.	Chemical Formula	C_3076_H_4656_N_818_O_883_S_24_
10.	Charge	Negative

* Extinction coefficients are in units of M-1 cm-1, at 280 nm measured in water.

**Table 4:** Active sites of human ACE.

Amino Acid	Position	Amino Acid	Position
Histidine	317	Alanine	318
Serine	319	Histidine	347
Glutamic Acid	348	Histidine	351
Glutamic Acid	375	Phenylalanine	421
Lysine	475	Phenylalanine	476
Histidine	477	Valine	482
Tyrosine	484	Tyrosine	487

**Table 5:** Inhibitors ranked on the basis of their S-values.

No.	Name	S-Values
1.	Teprotide	−20.1163
2.	Fosinopril	−18.9225
3.	Moexipril	−16.816
4.	Quinapril	−13.456
5.	Lisinopril	−12.502
6.	Cilazapril	−12.493
7.	Trandolapril	−12.2673
8.	Enalapril	−11.7516
9.	Ramipril	−11.3562
10.	Captopril	−10.8282
11.	Benazepril	−9.3245
12.	Perindopril	−8.105
13.	Allicin	−5.5448

**Table 6:** Lipinski’s rule of five for ADME analysis of our inhibitors (ligands)**.**

No.	Name	Lipinski’s Rule of Five					Drug-Likeness
		Molecular Weight (g/mol)	Lipophilicity (MLog *P*)	Hydrogen Bond Donors	Hydrogen Bond Acceptors	No. of Rule Violations	
		Less than 500 Dalton	Less than 5	Less than 5	Less than 10	Less than 2 Violations	Lipinski’s Rule Follows
1.	Teprotide	1101.26	−3.11	10	13	3: MW > 500, NH or OH > 5, N or O > 10, 1: MW > 500	No
2.	Fosinopril	563.66	3.74	1	7	0	Yes
3.	Moexipril	498.57	1.54	2	8	0	Yes
4.	Quinapril	438.52	2.17	2	6	0	Yes
5.	Lisinopril	405.49	−1.46	4	7	0	Yes
6.	Cilizapril	417.50	1.79	2	7	0	Yes
7.	Trandolapril	430.54	2.19	2	6	0	Yes
8.	Enalapril	376.45	1.32	2	6	0	Yes
9.	Ramipril	416.51	1.98	2	6	0	Yes
10.	Caprtopril	217.29	0.45	1	3	0	Yes
11.	Benzapril	424.49	2.23	2	6	0	Yes
12.	Perindopril	368.47	1.36	2	6	0	Yes
13.	Allicin	162.27	1.18	0	1	0	Yes
